# The shoot-feeding ecology of three *Tomicus* species in Yunnan Province, southwestern China

**DOI:** 10.1093/jis/14.1.37

**Published:** 2014-03-12

**Authors:** Jun Lu, Tao Zhao, Hui Ye

**Affiliations:** 1 Laboratory of Biological Invasion and Transboundary Ecosecurity, Yunnan University, Kunming 650091, China; 2 Ecological Chemistry Group, Department of Chemistry, Royal Institute of Technology, SE-10044 Stockholm, Sweden; + These authors contributed equally to the manuscript

**Keywords:** pine shoot beetles, *Pinus yunanensis*, population dynamics, shoot preference

## Abstract

Three *Tomicus* pine shoot beetles, *T. yunnanensis* (Kirkendall and Faccoli) (Coleoptera: Scolytidae), *T. minor* (Hartig), and *T. brevipilosus* (Wood and Bright), have been causing serious damage to Yunnan pine ( *Pinus yunnanensis* (Franchet) (Pinales: Pinaceae)) stands in Yunnan, southwestern China. However, their ability to coexist in the crowns of the same trees during the shoot-feeding phase has not been elucidated. In our study, we investigated and compared the shoot-feeding ecology of the three species of pine shoot beetle in *P. yunnanensis* in Anning County, Yunnan Province. Shoot-feeding by *T. yunnanensis*, *T. minor,* and *T. brevipilosus* initiated in April, May, and June, and ended in February, April, and May, respectively. Individual *T. yunnanensis* and *T. minor* adults fed in shoots for about seven months, and *T. brevipilosus* for nine months, before initiating reproduction. All three *Tomicus* species fed in the current-year shoots close to the apical bud. No specific overwintering behavior was observed prior to reproduction. The entrance hole of *T. yunnanensis* was furthest away from the apical bud, and *T. minor* was the closest to the apical bud. Differences in the spatial distribution of these shoot-feeding sites might reduce competition among the three beetle species. The long-lasting and overlapping shoot-feeding by the three *Tomicus* species may reduce the resistance of *P. yunnanensis* and facilitate the reproduction of these beetles in the trunks of living trees, and thus help explain the severe damage by *Tomicus* in *P. yunnanensis*.

## Introduction

The pine shoot beetles of the genus *Tomicus* (Latreille) (Coleoptera: Scolytidae) include eight species worldwide (Kirkendall et al. 2008; Li et al. 2010). Three species, *T. yunnanensis* (Kirkendall and Faccoli), *T. minor* (Hartig), and *T. brevipilosus* (Wood and Bright), have been reported causing damage to Yunnan pines ( *Pinus yunnanensis* (Franchet) (Pinales: Pinaceae)) in southwestern China (Ye and Ding 1999; Liu et al. 2010). More than 200,000 ha of *P. yunnanensis* in the Yunnan Province have been killed by *Tomicus* since the early 1980s (Ye and Ding 1999).

The life cycle of adult *Tomicus* bark beetles contains two phases, a reproduction phase and a maturation feeding phase. During the reproduction phase, sexually mature adults usually mate and lay eggs in the inner bark of trunks and the large branches of recently dead and weakened host trees. Larvae and pupae subsequently complete development and emerge as adults. Upon emergence, brood adults typically fly to the crowns of host pine trees, where they feed in the shoots and become sexually mature (Långström 1983; Ye 1991). Shoot-feeding by *Tomicus* can result in substantial growth loss (Långström and Hellqvist 1991; Eidmann 1992; Czokajlo et al. 1997), reduced tree resistance, and predispose these trees to subsequent reproductive attacks (Schlyter and Löfqvist 1990; Långström et al. 1993; Lieutier et al. 2003). Therefore, the shoot feeding is an important and damaging phase in the beetles’ life history.

Pine shoot beetles often infest host trees together. Two *Tomicus* species have been reported to attack pines in the same forest stands (Långström 1983; Ye and Ding 1999; Långström et al. 2002). For example, *T. piniperda* and *T. minor* often coexist in Scots pine ( *Pinus sylvestris* (L.)) stands (Långström 1983). Interspecific competition between co-existing species may be an important regulating factor in bark beetle population dynamics (Ye and Ding 1999). In addition, since different insect species may infest hosts in temporally and spatially different ways, their coexistence may lead to longer infestation time, increasing the damage done to the host. In Yunnan, the situation is more complicated: three *Tomicus* species ( *T. yunnanensis*, *T. minor*, and *T. brevipilosus*) are often observed attacking *P. yunnanensis* in the same stands, which represented a unique opportunity to investigate the coexistence pattern of *Tomicus*.

To study the shoot-feeding ecology of the three *Tomicus* and to understand their coexistence mechanisms during the shoot-feeding phase, we investigated the shoot-feeding period, population dynamics, and shoot-feeding preference of the three *Tomicus* species in *P. yunnanensis* for three consecutive years. The initial hypothesis was that the three *Tomicus* species would differ somehow in their ecological niches to reduce interspecific competition, and that their coexistence would prolong the shoot-feeding period and aggravate shoot damage to *P. yunnanensis*.

## Materials and Methods

### Study area

This study was carried out in a pure stand of ca 30-year-old *P. yunnanensis* at Qinglong Tree Farm (24.97°N, 02.33°E, 1800 m a.s.l) in Anning county, Yunnan Province. The heights of the experimental trees ranged from 7.5 m to 8.5 m and DBH of the trees ranged from 18 cm to 24 cm. The stand was about 300 ha in size with a density of about 900 trees/ha. The mean month temperature at the experimental site ranged from 9.6 to 22°C in the year, and its annual precipitation is about 900 mm. This pine stand has been attacked by pine shoot beetles since 1990. *T. yunnanensis*, *T. minor,* and *T. brevipilosus* have been observed attacking pines in this stand each year since 2004.

### Experimental procedures

Six trees in the stand were randomly chosen for investigation every month during the period from August 2006 to May 2009. Colors of the shoots in the experimental trees were carefully checked by investigators on the ground. The shoots with yellowish or brown needles near the shoot tip were considered to be evidence of *Tomicus* shoot-feeding. All infested shoots were cut using long-handled pruning shears and carried back to the lab. In the lab, the ages of the infested shoots were recorded based on the method described previously (Långström 1983; Ye 1994), the number of entrance holes per shoot were counted, and the diameter of the shoot at each entrance hole and the distance from the entrance hole to the shoot tip were measured using a vernier cali-per (HMCT 6202-01,), but only for infested shoots collected during June 2007 to May 2008. After the above measurements, the shoots were dissected. All the beetles collected from these shoots were identified under a stereoscope (smzl 500, Nikon Instrument, www.nikon.com) (Kirkendall et al. 2008). The number of each *Tomicus* species corresponding to the damaged shoots was recorded.

To confirm the commencement and termination of the shoot-feeding period, the trunks of all the pine trees along the forest edge were checked monthly for *Tomicus* infestation from October to June in 2006–2008. Three infested pine trees were chosen randomly and cut down every month. The outer bark of the main trunk for these trees was removed. All brood adults were collected and taken back to the lab, where they were identified under a stereoscope. The number of each *Tomicus* species was recorded. Duration of the shoot-feeding period for each of the three *Tomicus* species was defined based on the appearance of these beetles in the trunks, which is where they breed and develop.

### Data analysis

The differences in shoot diameters at the entrance holes and the distances from entrance hole to the shoot apex between three *Tomicus* species, for shoots collected during June 2007 to May 2008, were compared by the Kruskal-Wallis test. If treatments were significantly different ( *P* < 0.05), means were separated using the Mann-Whitney test at *P* = 0.05 (SPSS 13, SPSS Inc., www.spss.com).

## Results

### Shoot-feeding period

**Figure 1.**
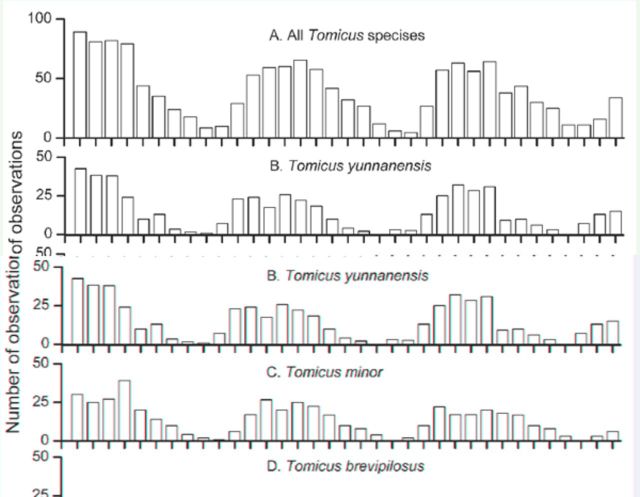
Number of (A) all *Tomicus* species, (B) *T. yunnanensis*, (C) *T. minor*, and (D) *T. brevipilosus* observed in the shoots of six *Pinus yunnanensis* trees at Anning, Yunnan Province, each month during the period from August 2006 to June 2009. The x-axis denotes the month for investigation in years 2006–2009, and the yaxis denotes the number of beetles observed in the relevant month. High quality figures are available online.

All three *Tomicus* species were observed shoot-feeding on *P. yunnanensis* during almost all months of the year (Figure 1). In winter, they continued feeding inside the shoots or entered the trunks of *P. yunnanensis* for breeding. No *Tomicus* adult departed the shoot to overwinter at the base of the trees as they do in the USA and northern Europe (Långström 1983; Ye et al. 2002).

The timing of initial shoot-feeding by newly-emerged *Tomicus* brood adults and the duration of shoot-feeding throughout the year varied by species (Figure 1). Shoot-feeding by *T. yunnanensis* brood adults started in April, followed by *T. minor* in May, and *T. brevipilosus* in June. The main shoot feeding period of *T. yunnanensis* ended in November, evidenced by reproduction found in the trunks (Figure 1B). The last fully-mature *T. yunnanensis* brood adults were found in shoots in February. The main flight period during which *T. yunnanensis* adults departed the shoots for breeding sites was from late November to the middle of February. Similarly, the main flight period for reproduction was initiated in December and ended in April for *T. minor* (Figure 1C), and occurred during April and May for *T. brevipilosus* (Figure 1D). Based on these observations, the shoot-feeding period was from April to February for *T. yunnanensis*, from May to April for *T. minor*, and from June to April for *T. brevipilosus*.

However, the entire shoot-feeding period of the three *Tomicus* species was not equal to the duration of shoot-feeding by a single adult for any *Tomicus* species. In this study, the duration of shoot feeding by a single adult was defined as the period from the first newly-emerged adult observed in the shoot to the first entrance hole observed in the trunk. A single *T. yunnanensis* or *T. minor* adult fed in shoots of *P. yunnanensis* for about seven months, and a *T. brevipilosus* adult for about nine months.

### Population dynamics

Though *Tomicus* adults fed in the shoots of *P. yunnanensis* throughout the year, their numbers varied extensively with the season of the year. In general, the number of adults found in the shoots increased from April to July, remained at relatively high levels from August to October, and then gradually decreased from November to March (Figure 1A). However, some differences were observed between beetle species. For example, the population of *T. yunnanensis* feeding in the shoots gradually increased from May to June or July, remained at relatively high levels until October or November, and then fell to low levels in March or April (Figure 1B). For *T. minor*, the shoot-feeding population gradually increased during June to July/August, remained at high levels from July/August to November/December, and then decreased at a relatively slow rate (Figure 1C). Similarly, *T. brevipilosus* populations increased rapidly in July, remained at high levels from July to February, and then decreased to low levels in May (Figure 1D). Overall, the bell-shaped population curve of *Tomicus* in the shoots reflected the movement of brood adults into the crowns as they emerged from the breeding materials during spring and summer, and then departing the crowns in autumn and winter, when they initiated breeding after their sexual maturation (Figure 1).

### Shoot-feeding preference

**Figure 2.**
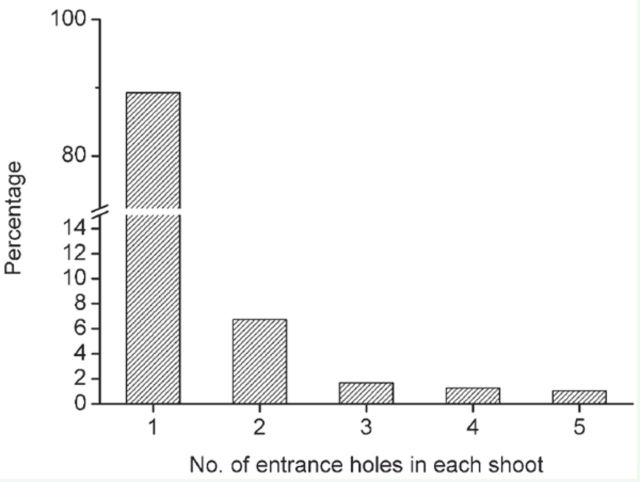
The percentages of the infested shoots with one to five entrance holes in each shoot. The infested shoots (n = 1563) were collected from *Pinus yunnanensis* at Anning, Yunnan Province, during 2006–2009. High quality figures are available online.

At Anning, *Tomicus* were only found shoot-feeding in current-year shoots. Most infested shoots only had one entrance hole, but some shoots contained two to five entrance holes in each shoot. Over the 35-month span of our study, in total 1563 infested shoots were examined. 89.2% of these shoots contained only one feeding tunnel, 6.0% of the shoots contained two, and 4.8% of the shoots contained three or more tunnels in each shoot (Figure 2). In the 169 shoots with two or more feeding tunnels, 67.5% of them were infested with only *T. brevipilosus,* 16.6% of them infested with *T. yunnanensis* and *T. brevipilosus,* 10.0% of them infested with *T. yunnanensis* and *T. minor*, and 5.9% infested with *T. minor* and *T. brevipilosus.* All pairwise combinations of the three beetle species were found tunneling in the same shoot, but never were all three species found in the same shoot.

**Figure 3.**
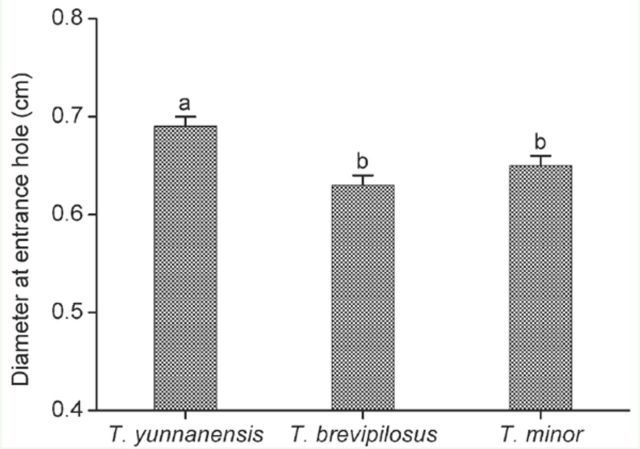
Mean diameter of *Tomicus*-infested shoots of *Pinus yunnanensis* as measured at the entrance holes. The infested shoots were collected from Anning, Yunnan Province, from June 2007 to May 2008. Data are expressed as means ± 1 SE, n = 119, 158, and 155 for *T. yunnanensis*, *T. brevipilosus,* and *T. minor* respectively. Bars with different letters are significantly different at *P* < 0.05, based on Mann-Whitney test. High quality figures are available online.

To compare the shoot-feeding preference of three *Tomicus* species in *P. yunnanensis,* the shoot diameter was measured at the entrance hole for each beetle species. Overall, *T. yunnanensis* were observed feeding in shoots within a diameter range of 0.4–1.3 cm, and 81.5% of the attacks occurred in the shootswith a diameter of 0.6–0.8 cm. *T. minor* and *T. brevipilosus* seemed to prefer shoots ofsimilar thickness; 76.1% of *T. minor* tunnels similar among the three species, however, statistically the shoots attacked by *T. yunnanensis* were significantly larger than the shoots attacked by *T. brevipilosus* and *T. minor* (Figure 3).

**Figure 4.**
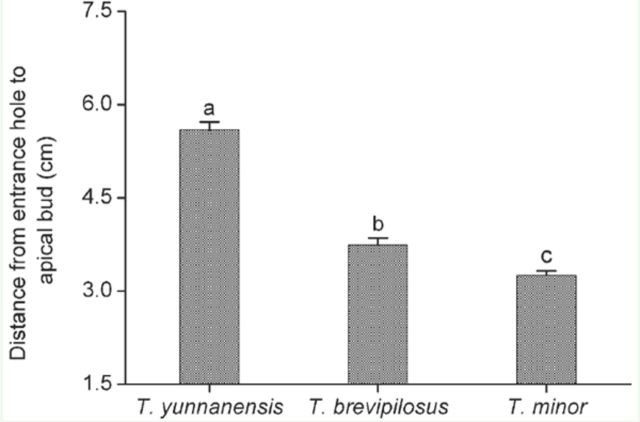
Mean distance from the entrance holes of *Tomicus* shoot-feeding tunnels to the apical buds in the shoots of *Pinus yunnanensis.* The infested shoots were collected from Anning, Yunnan, from June 2007 to May 2008. Data are expressed as means ± 1 SE, n = 119, 158, and 155 for *T. yunnanensis*, *T. brevipilosus,* and *T. minor* respectively. Bars with different letters are significantly different at *p* < 0.05, based on Mann-Whitney test. High quality figures are available online.

The position of the entrance hole was defined as the distance from the entrance hole to the apical bud. For *T. yunnanensis*, the mean distance from the entrance hole to the apical bud was 5.02 ± 0.13 cm, which was significantly longer than that of 3.7 ± 0.11 cm for *T. brevipilosus* ( *P* < 0.01), and 3.2 ± 0.07 cm for *T. minor* ( *P* < 0.001). In addition, the entrance hole of *T. minor* was closer to the apical bud relative to *T. brevipilosus* ( *P* = 0.032) (Figure 4)

## Discussion

In Europe and the USA, *T. piniperda* and *T. minor* overwintered at the base of the host trees in which they had fed in the shoot (Kauffman et al. 1998; Fernández et al. 1999; Haack et al. 2001; Poland et al. 2003). By contrast, in Yunnan Province, the three *Tomicus* species did not overwinter at the base of the tree, but rather remained in the shoots of *P. yunnanensis* until they initiated breeding in the main trunks of *P. yunnanensis*. In northern Europe, the spring flight activity of overwintering *Tomicus* starts when the daily maximum temperature exceeds 10–12°C (Långström 1983). In Yunnan, *T. yunnanensis* successfully reproduced at a temperature of 10°C (Ye 1994; Ye and Bakke 1997).In the present study area, the mean daily air temperature in the coldest months, January and February, is about 9.6°C. The mean daytime air temperature during this period is around 16.3°C, which is sufficiently warm to support the normal activities of *Tomicus*. Similarly, *T. destruens* in southern Europe spends winters in the shoots because it is usually warm in winter months (Faccoli 2007, 2009). These observations suggest that the overwintering behavior of *Tomicus* adults is regulated in large part by local temperature conditions and may have a few genetic components (Poland et al. 2002; Ye et al. 2002).

Pine shoot beetles spend longer periods of time in shoots in Yunnan than in northern Europe or North America (Ye 1991, 1994; Ye and Lieutier 1997; Haack et al. 2000, 2001; Långström et al. 2002). In Sweden, the shoot-feeding period by a single *T. minor* adult lasted 105 days, during which time it fed in one to two shoots (Långström 1983). By contrast, a *T. yunnanensis* adult usually con-sumes four to six shoots during its seven-month shoot-feeding period (Ye 1996). Intensive shoot-feeding by *Tomicus* reduces tree growth (Långström and Hellqvist 1991; Eidmann 1992; Czokajlo et al. 1997) and renders the trees susceptible to subsequent trunk attacks (Schlyter and Löfqvist 1990; Långström et al. 1993; Lieutier et al. 2003). The severe shoot damage caused by the long lasting and overlapping shoot-feeding of the three *Tomicus* might reduce the resistance of *P. yunnanensis* to a low level and thus facilitate the reproduction of *Tomicus* in the trunks of living trees.

The average distance from the entrance holes of *T. yunnanensis* to the apical bud was statistically longer than that of the other two *Tomicus* species. Additionally, these three *Tomicus* species initiated shoot-feeding at different times. *T. yunnanensis* started shoot-feeding one month earlier than *T. minor* and two months earlier than *T. brevipilosus*. The three *Tomicus* species differed temporally and spatially in their shoot-feeding propensities, which may lower interspecific competition during shoot-feeding.

This study compared the shoot-feeding ecology of three *Tomicus* species in *P. yunnanensis*. The varied spatial and temporal distribution of the three *Tomicus* species during shoot-feeding may reduce interspecific competition and thus enhance their coexistence. Our study, hence, improves understanding of the coexistence mechanisms and the ecology of *Tomicus* in Yunnan during their shoot-feeding phase. However, an integrated study considering both shoot-feeding and trunk attack should be conducted to more deeply understand the interactions among the three *Tomicus* species.
